# Unilateral biportal endoscopy: review and detailed surgical approach to extraforaminal approach

**DOI:** 10.1530/EOR-24-0137

**Published:** 2025-03-03

**Authors:** João Pedro Gomes Reis, Eduardo Moreira Pinto, Artur Teixeira, Ricardo Frada, Diogo Rodrigues, Raquel Cunha, António Miranda

**Affiliations:** ^1^ Unidade Local de Saúde de Trás-os-Montes e Alto Douro, Vila Real Serviço de Ortopedia e Traumatologia, Vila Real, Portugal; ^2^ Unidade Local de Saúde Entre Douro e Vouga, Santa Maria da Feira Serviço de Ortopedia e Traumatologia, Unidade de Coluna, Santa Maria da Feira, Aveiro, Portugal; ^3^ Unidade Local de Saúde do Porto, Porto Serviço de Ortopedia e Traumatologia, Porto, Portugal

**Keywords:** spine, endoscopy, biportal, foraminal

## Abstract

- Foraminal and extraforaminal lumbar disc herniations are common sources of pain and disability. Classic surgical treatments, such as open approach through Witsel technique, often involve resection of the superior articular process to decompress the foraminal space.
- Unilateral biportal endoscopy (UBE) has emerged as a minimally invasive alternative, providing enhanced visualization and precision while minimizing soft tissue damage.
- The extraforaminal approach using UBE offers a more effective solution for extraforaminal herniations, requiring less bone resection, reducing the risk of pars fracture and enhancing visualization.
- This article presents a comprehensive methodology for the extraforaminal approach, supported by an illustrated guide, surgical tips and highlights of UBE’s advantages over traditional techniques.

## Introduction

Lumbar disc herniation (LDH) is a common cause of pain and disability. The most commonly used classification system is based on its axial localization – central, paracentral and lateral. Concerning lateral disc herniation, it can be divided into foraminal and extraforaminal types – accounting for 0.7–11.7% of all LDH (1).

In addition to the compression of the exiting nerve root and dorsal root ganglion, which is typically associated with severe pain and worst surgical outcomes, it also presents some particularities in the surgical approach.

A Witsel approach is most commonly used to access the facet joint and transverse process, with amplification in the intertransverse fascia, and the nerve root can be identified and traced back to the foramen (2). In case of foraminal herniation, it can be necessary to remove part of the superior articular process (SAP) to achieve proper disc access.

Nowadays, unilateral biportal endoscopy (UBE) offers a minimally invasive solution with enhanced visualization and precision.

UBE is usually performed through an interlaminar route, which can provide access to foraminal LDH with facet joint resection. It has been shown that facet joint resection is related to spinal stability (3).

UBE has been employed at our institution instead of microscopic surgery to address most of LDH.

The purpose of this study is to review the literature on extraforaminal approaches using UBE and report our technique.

## Methods

### Literature search

The PubMed library database was utilized in the development of this literature review. A comprehensive search was completed collecting all articles dated before May 31, 2024. The search terms used were as follows: ‘biportal’ and ‘hernia’ and (‘foraminal’ or ‘extraforaminal’).

A total of four articles were identified and will be discussed; they are summarized in Table 1.

**Table 1:** Summary of selected articles.

Reference	Sample size, *n*	Study type	LOE	Technique description
Park *et al.* (4)	35	Descriptive/observational	IV	Good description, instructive images (only L5–S1)
Park *et al.* (5)	X	Case report	IV	Good description, instructive images (only L5–S1)
Heo *et al.* (6)	14	Descriptive/observational	IV	Resumed description, no images (only far-out syndrome)
Ahn *et al.* (7)	21	Descriptive/observational	IV	Resumed description, no images – video in description

LOE, level of evidence.

### Surgical technique

**Figure 1:**
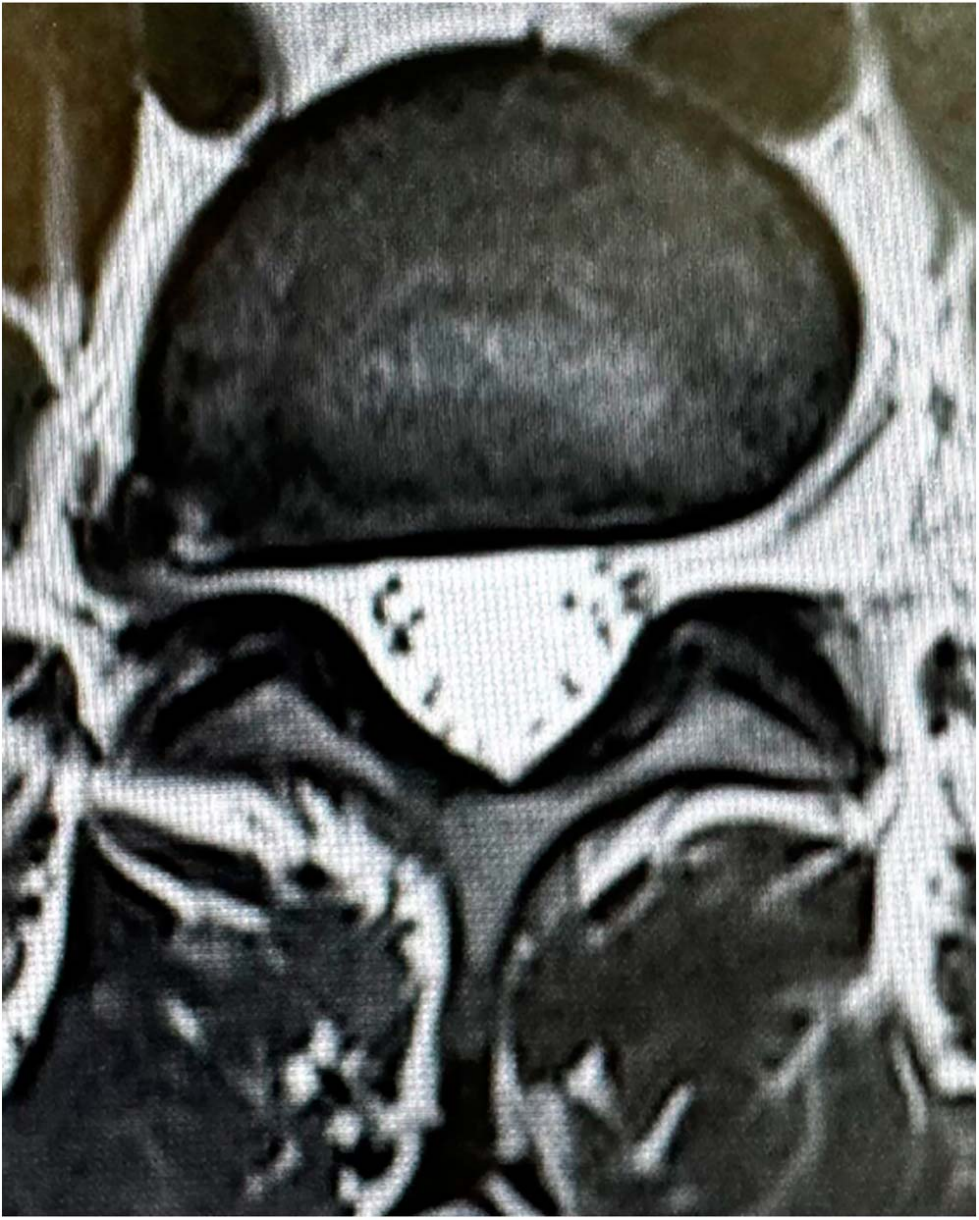
Axial cut of lumbar magnetic resonance (T2) with an extraforaminal hernia of L4–L5 disc compressing the exiting nerve root at right side.

**Figure 2:**
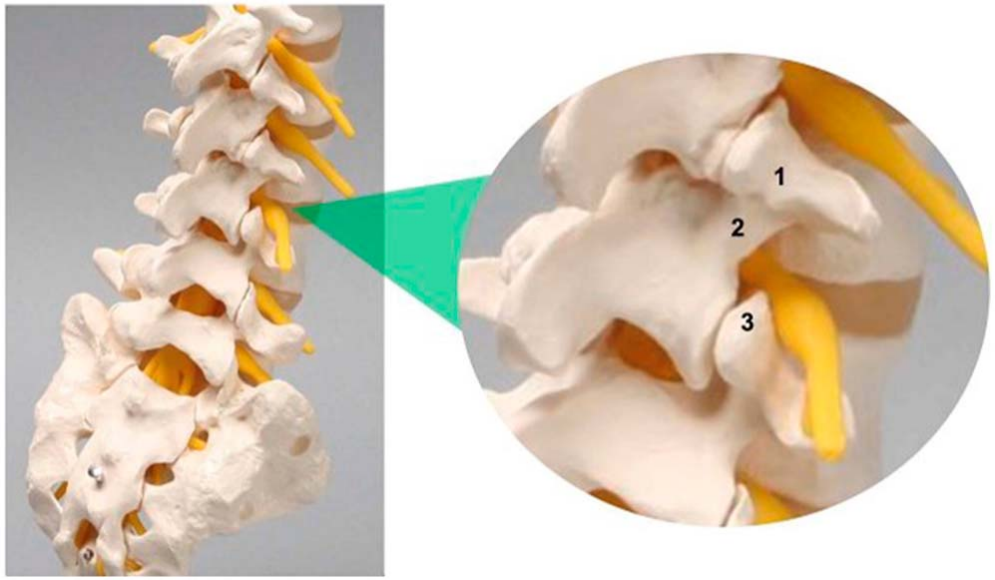
Sawbone model of lumbar spine with identification of essential landmarks to perform a paraspinal UBE. 1 – superior transverse process; 2 – lamina isthmus; and 3 – SAP inf.

To describe the paraspinal technique, we present a case of an extraforaminal hernia L4–L5 on the right side (Fig. 1). For the decompression of the extraforaminal and foraminal spaces, three bony landmarks are essential – the superior transverse process, the isthmus and the SAP of the inferior vertebra (Fig. 2).

#### Skin marking

**Figure 3:**
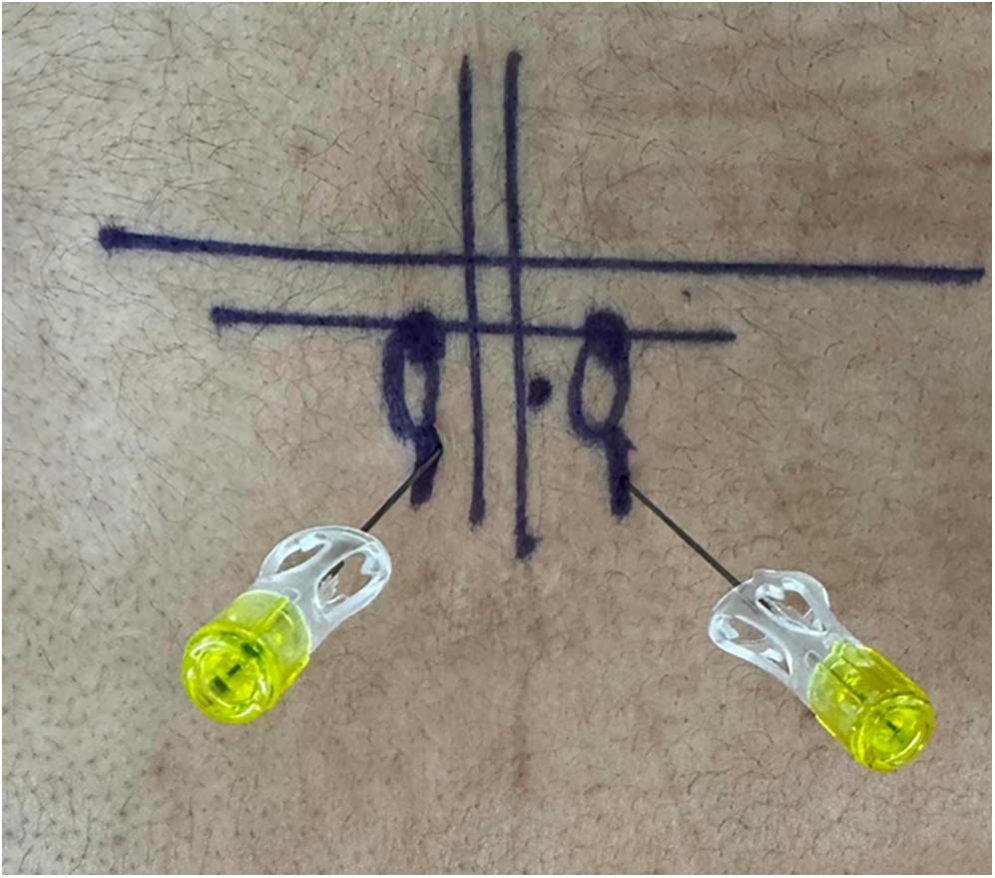
Landmarks for paraspinal approach.

The landmarks are marked as in the interlaminar approach on the anteroposterior view of the C-arm fluoroscopy. Therefore, the superior and inferior disc limits of the corresponding level, the pedicular line and the superior and inferior pedicle should be marked (Fig. 3).

**Figure 4:**
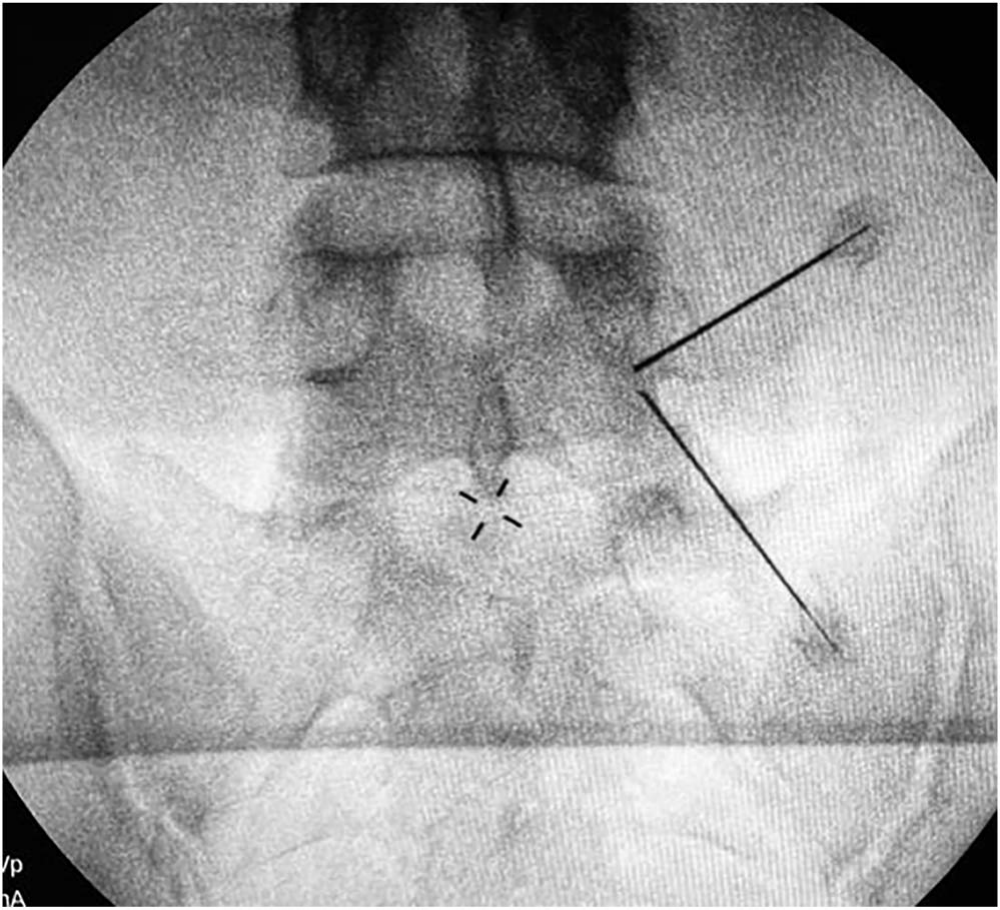
AP X-ray with needles to confirm proper docking point placement.

Our docking point will be at the isthmus of the lamina, which should be marked superficially for guidance (Fig. 4). The first portal is marked corresponding to the transverse process, and the second should be approximately 3 cm caudal to the first. Portal incisions are made in a horizontal way within 2–3 cm distance from the pedicular line.

Using fluoroscopic controls, dilators are inserted into the docking point – the isthmus– through both portals – camera and working. The dilation phase should also be used to create Son’s space through blunt multifidus dissection.

#### Soft tissue and bone working

**Figure 5:**
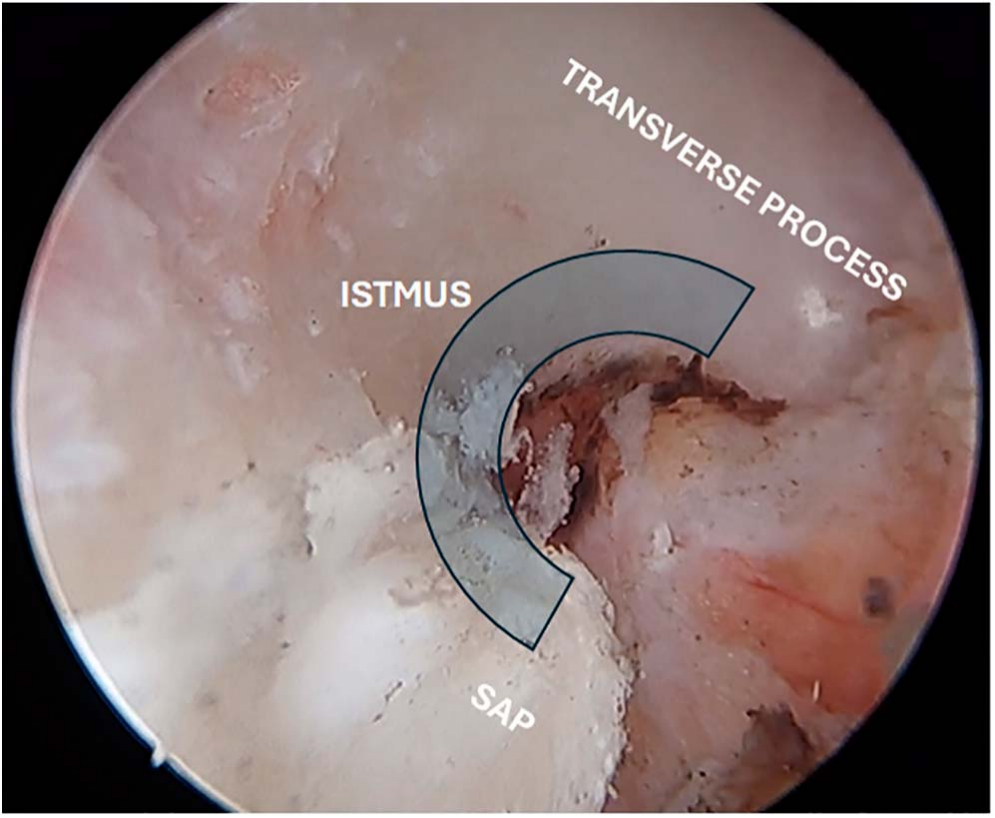
Bony landmarks after proper debridement – the “C” sign results of proper debridement exposing our landmarks for proceeding with bone work.

After successful docking, we should clear soft tissue using radiofrequency or shaver to clearly identify the transverse process, the isthmus and the SAP (Fig. 5).

Using a high-speed drill and Kerrison rongeur, a laminotomy of the lateral portion of the lamina and a SAP partial facetectomy are performed. Care should be taken not to remove too much facet joint, avoiding pars fractures and subsequent instability. It is important to have in mind vascular anatomy, since facet joint artery, a branch of lumbar segmental artery, can be lesioned in this step and cause bleeding of difficult control.

After foraminoplasty, the ligamentum flavum should be removed in order to expose the exiting nerve root and lumbar disc. After identification of the nerve root, a complete decompression should be performed according to the compression identified – osteophytes, disc herniation or far-out syndrome at lumbosacral transition.

A foraminal hernia demands a more extensive bone work than an extraforaminal hernia, but the amount of bone removal is not sufficient to cause instability with proper technique.

**Figure 6:**
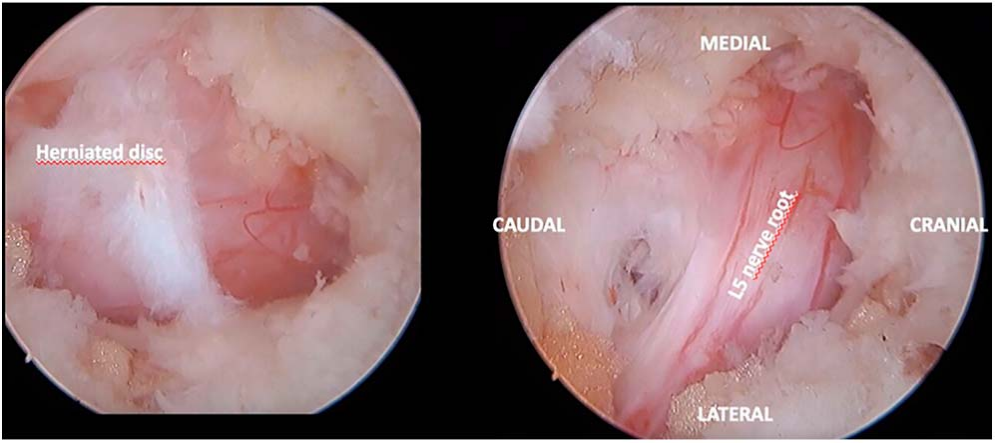
Identification of the herniated disc compressing the exiting nerve root (A); decompressed nerve root after discectomy (B).

At lumbar disc level, an identification of the emerging nerve root and subsequent discectomy should be performed. If necessary, the identification of the descending nerve root and lateral recess decompression is also possible (Fig. 6).

**Figure 7:**
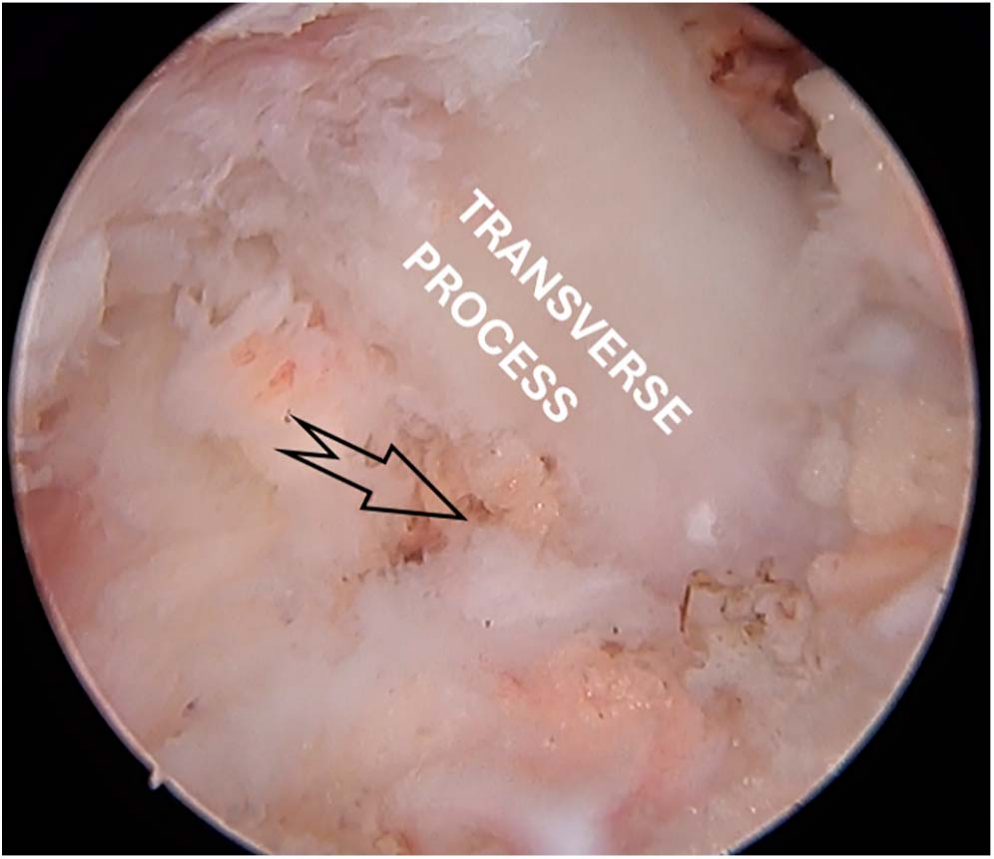
Paraspinal approach with identification of transverse process with the intertransverse ligament on inferior-lateral border.

#### Pearls

There are three fundamentals that the surgeon must have while performing a paraspinal approach.

Iatrogenic retroperitoneal fluid retention can be a complication of the paraspinal approach. Usually, it only causes abdominal distention and discomfort and has spontaneous reabsorption. To avoid it, pressures above 30 mmHg should be avoided and transverse process should be respected as our deep limit, since violation of the intertransverse ligament is a risk factor for this condition (Fig. 7).

Intraforaminal lesions are more easily addressed with an angled material – curette, Kerrison and pituitary rongeurs.

Although triangulation is helpful in the first case to assess proper angulation, it should be performed systematically at level L5–S1 to prevent iliac crest conflict. In the presence of conflict, it can be managed with 1-cm medialization of the distal portal.

## Discussion

UBE is mostly performed through an interlaminar route that offers proper visualization for most LDH without extensive bone work.

There are few reports in the literature for accessing foraminal LDH with UBE. There are three different documented routes: interlaminar ipsilateral, sublaminar and extraforaminal.

The ipsilateral approach has the disadvantage of facet joint resection with more traction on the nerve root (8). This is an important factor since the ipsilateral approach has a higher resection rate of the articular process (22.6%), increasing the fracture risk of the inferior articular process by 6% (9).

The contralateral approach allows more precise identification of the middle line of the spinal canal and facet joint preservation. However, the surgeon should overcome a steep learning curve and have already some expertise since this will demand more bone and soft tissue work. In addition, the surgical path is too long and LDH may not be clearly identified, making this not suitable for novice surgeons. Meningeal irritation can be found in longer surgeries due to excessive saline irrigation.

Concerning the literature review, three of the four articles describe decompression of L5–S1. Only Ahn *et al.* briefly described a paraspinal approach for LDH but made the article more complete with exemplification videos.

Zhu *et al.* reported in a similar way an extraforaminal approach with incisions 2 cm lateral to the pedicle of L5–S1 and 1.5 cm above and below the L5 isthmus. This case was an adjacent segmental disease with prior instrumentation and decompression (10).

Park *et al.* documented a similar way of approaching extraforaminal space through Son’s corridor that is limited by the nerve root, transverse process and lumbosacral ligament – enabling the removal of lumbosacral ligaments. It offers the advantage of less manipulation of the nerve root, but it is only valid for lumbosacral transition (4).

UBE paraspinal approach has several advantages over the microscopic paraspinal approach – less muscular and bone damage – which is a less harmful and easier way to approach foraminal space with less postoperative back pain and to return to daily activities earlier (11, 12).

There are also several technical advantages over other techniques. The proper illumination and magnification make assessing the anatomical details easier. The application of a continuous flow of irrigation fluid reduces fogging, helps control bleeding and enhances the lysis of adherences, and some studies advance it as a hypothesis to reduce the infectious risk (13).

The described approach also presents advantages over conventional UBE approaches, presenting less bone and soft tissue work, which implies less risk of surgical complications and reduced surgical time. It is also superior due to minimal resection of the SAP, less risk of instability and the maintenance of the interlaminar tissue intact if there is a need to perform a revision or a different kind of procedure.

## Conclusion

Currently, there is a paucity of data concerning the extraforaminal approach to lumbar spine with UBE.

To our knowledge, this is the first technical report that summarizes this technique.

Therefore, UBE is a reproducible technique that can effectively treat foraminal and extraforaminal pathology through an extraforaminal approach.

## ICMJE Statement of Interest

The authors declare that there is no conflict of interest that could be perceived as prejudicing the impartiality of the work reported.

## Funding Statement

This work did not receive any specific grant from any funding agency in the public, commercial or not-for-profit sector.

## Author contribution statement

Each author contributed individually and significantly to the development of this article. JPGR designed the study and elaborated the article. EMP, a surgeon, substantially contributed to the conception and revision of this article. AT, a surgeon, substantially contributed to the conception and revision of this article. RF, a surgeon, substantially contributed to the conception and revision of this article. DR was involved in photography documentation and elaboration of the article. AM revised the article and approved the final version of the manuscript.
